# Human IgM monoclonal antibodies block HIV-transmission to immune cells in cervico-vaginal tissues and across polarized epithelial cells *in vitro*

**DOI:** 10.1038/s41598-018-28242-y

**Published:** 2018-07-05

**Authors:** Claudia Devito, Rada Ellegård, Tina Falkeborn, Lennart Svensson, Mats Ohlin, Marie Larsson, Kristina Broliden, Jorma Hinkula

**Affiliations:** 1 Linköping University, Department of Clinical and Experimental Medicine, Division of Molecular Virology, 58185 Linköping, Sweden; 2 Department of Virology, Swedish Institute for Infectious Disease Control, 17157 Solna, Sweden; 3 HD Department of Clinical Virology, Stockholm, Sweden; 4 Department of Immunotechnology, Lund University, Lund, Sweden; 5 Department of Medicine Solna, Unit of Infectious Diseases B2:00, Center for Molecular Medicine, Karolinska University Hospital, Karolinska Institutet, 171 76 Stockholm, Sweden

## Abstract

The importance of natural IgM antibodies in protection against infections is still emerging and these antibodies have a potential role in the maintenance of homeostasis through clearance of apoptotic bodies, complement-dependent mechanisms, inflammation and exclusion of misfolded proteins. Natural IgM act as a first line of defence against unknown hazardous factors and are present in most vertebrates. We investigated the functional capacity of anti-HIV-1 IgM monoclonal antibodies, from a combinatorial Fab library derived from healthy individuals, and evaluated their protective role in inhibiting HIV-1 *in vitro* when passing across the human mucosal epithelial barrier. Primary HIV-1 isolates were efficiently transmitted over the tight polarized epithelial cells when added to their apical surface. Efficient inhibition of HIV-1 transmission was achieved when anti-HIV-1 IgM monoclonal antibodies were added to the basolateral side of the cells. Two of these human IgM MoAbs had the ability to neutralize HIV and reduced infection of dendritic cells in primary cervico-vaginal tissue biopsies *in vitro*. This indicates a potential role of natural IgM antibodies in the reduction of HIV-1 transmission in mucosal tissues and improve our understanding of how natural IgM antibodies against a neutralizing epitope could interfere with viral transmission.

## Introduction

The establishment of HIV infection in the mucosa during sexual transmission is dependent on the virus ability of the virus to overcome layers of innate barriers/responses and find HIV target cells where the infection can be initiated. One important component of the innate responses is natural IgM (nIgM) antibodies. Our understanding of the role of these antibodies in the protection against infections is emerging; they function as the first line of defence in neutralizing invading pathogens, and they facilitate T-cell recognition, B-cell isotype switching, and the transport of antigens to lymphoid tissue^1^. Moreover, nIgM participate in tissue homeostasis by regulating inflammatory processes and autoimmune diseases^2^. IgM is generated at very early stages in B-cell ontogeny and is produced and secreted without antigen stimulation. Its pentameric structure allows the binding of multiple antigenic sites in both the systemic and mucosal compartments^3–5^. The circulating nIgM is polyreactive, whereas other immune IgM clones have specific antigen binding affinities^6–8^.

The main activity of nIgM is to facilitate the complement pathway, and there are enormous property variations among the IgM antibodies even if they have the same binding specificity. Mucosal tissue-associated B-lymphocytes produce IgA^9^, but they also produce IgM and IgG, and most of them produce the J chain polypeptide^10^. IgM antibodies bind to two receptors known as Fcα/uR and polymeric Ig receptor^11,12^. The latter mediates transport through the mucosal epithelium of secretory IgM (S-IgM). Consequently, the antibodies present in mucosal secretions can bind directly to external proteins on the viral surface, preventing attachment to the epithelium, or interact with and destroy the virus within the epithelial cell^13–15^. It has been shown that HIV-1 can penetrate a tight epithelial cell barrier^16^, IgA and IgM from colostrum and cervico-vaginal fluids from HIV-1-positive women neutralize HIV-1 transport across mucosal surfaces^17,18^.

Several human IgM-derived monoclonal antibodies (MoAbs) obtained from the blood, lymph nodes, and spleens of healthy donors showed high-avidity binding to gp120 derived from HIV isolates from different clades. These monoclonal antibodies selected from a large phage-displayed naive human antibody library did not neutralize CCR5 primary isolates, although weak neutralization was detected against all CXCR4 isolates tested^19,20^. In HIV infection, antibodies against conserved epitopes need to develop early to allow the early humoral recognition of a broad HIV strain repertoire. However, most antibodies that react with gp120 bind to its variable regions and unfortunately only rarely to the conserved HIV epitopes, which display poor immunogenicity^21^. The third variable loop region is one of the earliest identified regions containing neutralizing B cell epitopes and several studies have shown that the “tip” of the V3-region in the HIV-1 outer envelope of HIV-1 gp120 is relatively conserved^22,23^.For that reason, did we in a previous study, select B cells with natural production of IgM MoAbs recognizing the HIV-1 V3 region from HIV negative healthy individuals. The selected B cells were turned into immortalized cell lines. Interestingly, several anti-V3 IgM clones were detected, with the epitope recognition of the amino acid sequence 294–323, which contain the most conserved amino acids G-P-G V3-tip sequence in the HIV V3 region. They differed in avidity index, and in the size of the epitopes mapped^20^. Moreover, a typical and highly relevant property with IgM antibodies is their relatively weak epitope-binding affinity, but their multi-valency still develop a fair avidity or “stickiness” against a target protein such as gp120. This property make IgM molecules interesting to study, but hard to investigate in their detailed fine-epitope-specificity.

This HIV-1 envelope protein surfaces containing gp120/gp41 have been used as a target in experimental vaccine protocols. Antibodies against the CD4-binding domain (CD4BD) of gp120 are produced in small amounts in the pool of neutralizing antibodies in infected individuals, whereas others do not have any neutralizing activity at all even after CD4BD binding^24^. It has been postulated that the failure to induce broad neutralizing antibodies ensues from differences in the molecular structures of monomeric and native gp120^25^. However, oligomeric gp120 also fails to produce broad neutralizing antibodies^26^. Nevertheless, antibodies against HIV gp120 amino acid residues 421–433 that are present before viral exposure have been shown to neutralize different HIV subtypes^27^.

The aim with this study was to establish if the naturally occurring IgM mAbs we previously developed had antiviral efficacy against primary HIV-1 isolates so the ability of four HIV-1 gp120-binding human IgM monoclonal antibodies derived from healthy individuals to block HIV-1 was analysed. We also determined whether the viruses remained infectious during transport within human cervical mucosa biopsies from healthy donors and the human intestinal epithelial cell line. The formation of Ag-IgM complexes could be one of the mechanisms responsible for the enhancement of humoral immune responses and could be a key component for prophylactic mucosal HIV intervention strategies.

## Results

### Transcytosis of IgM antibodies and HIV-1 across the polarized epithelial cell membrane

**Figure 1:**
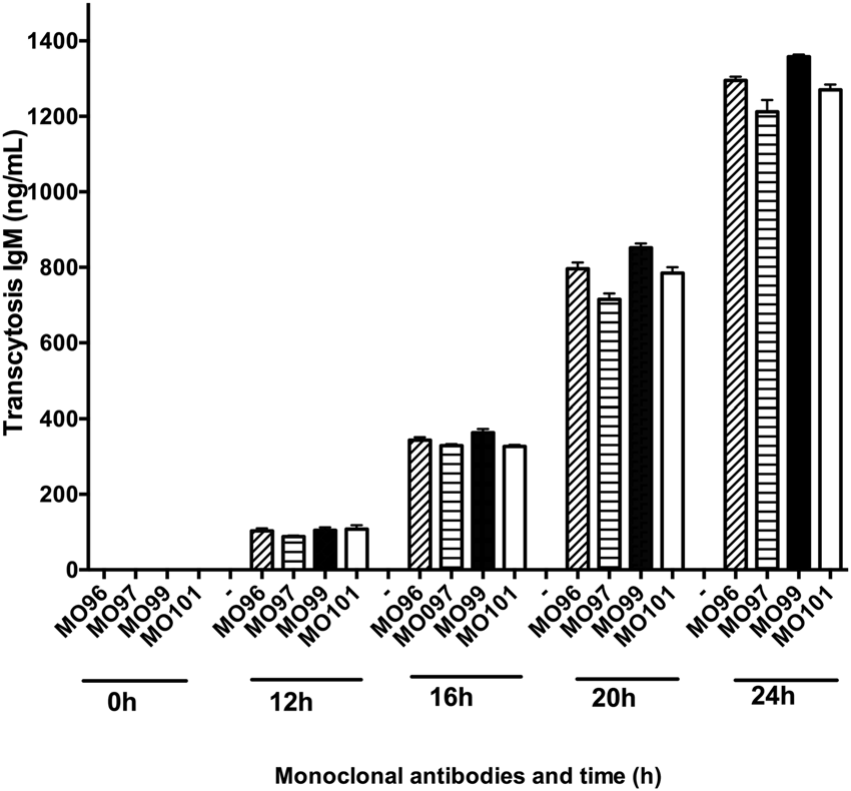
Transcytosis of HIV-1 from the apical to the basolateral side of the polarized epithelial cell membrane. The kinetics of the HIV-1 envelope specific human IgM MoAbs MO96, MO97, MO99 and MO101 transcytosis from the basolateral to apical side in the Transwell cell culture system using the human epithelial cell line Caco-2-pIgR+. The levels of transcytosed IgM MoAbs in the apical medium over the *24* *h* study period was assessed by gp160 MoAb reactive ELISA. Shown are median IgM transcytosis activity (Median and range). N = three separate experiments/monoclonal antibody (MoAb).

**Figure 2:**
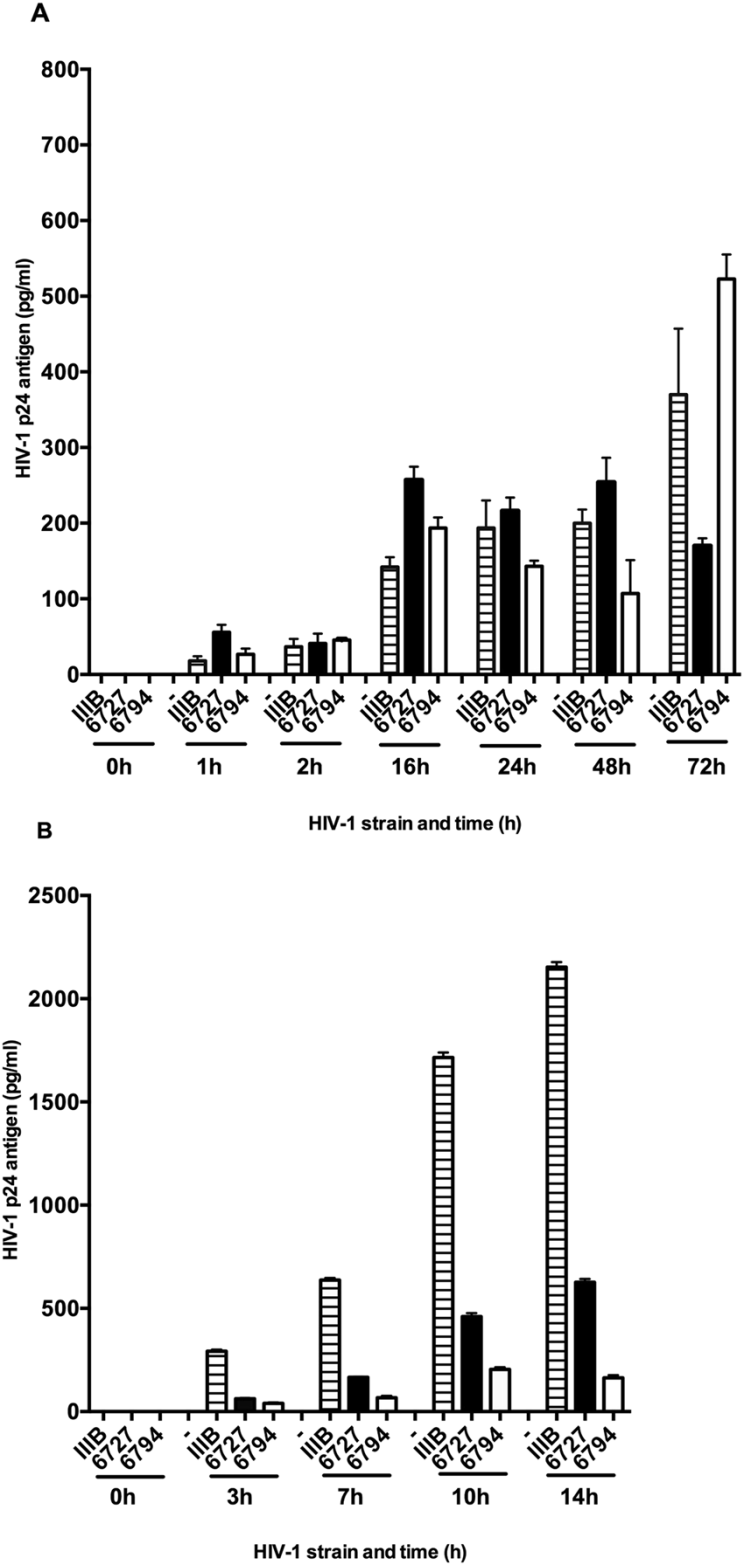
Transcytosis of HIV-1 from the apical to the basolateral side of the polarized epithelial cell membrane. Three HIV-1 isolates, the laboratory adapted HIV-1 IIIB/Lai strain and two primary HIV-1 clade B isolates, one non-syncytia-inducing strain NSI6727, and one syncytia-inducing strain SI6794, were added to the apical side of the Transwell cell culture system human epithelial cell line Caco-2-pIgR+. (**A**) Culture supernatants were collected from the basolateral side over a 72-h period and tested for HIV-1 p24 by ELISA. The median and interquartile ranges of HIV-1 p24 antigen concentration from two to three analyses/HIV-1 isolate are shown. (**B**) Infectivity of the transcytosed HIV-1 collected at the basolateral side was measured by the ability to infect human target cells *in vitro*. Jurkat T cells was used for HIV-1IIIB/Lai and IL-2/PHA-activated human PBMCs for the two primary HIV-1 clade B isolates. Basolateral supernatant with HIV-1 was incubated with 10^6^ target cells for 24 hours before washed and cultured. Supernatants were collected over a 14 h period and the infection levels assessed by HIV-1 p24 antigen-specific ELISA.

The properties of the propagated human IgM antibodies were first analysed by their transcytosis abilities. A Transwell model system was used to measure the epithelial transmembrane transport of the IgM monoclonal Abs using human Caco-2-pIgR+ cells, which are known to facilitate HIV-1 virus transcytosis *in vitro*^16,18^. The kinetics of IgM transcytosis from the basolateral medium to the apical side of the polarized epithelial Caco-2-pIgR+ cells was assessed and at 24 h the IgM levels reached 1283 ng/mL (median 1284 ng/mL with range 1190–1362) (Fig. 1). Caco-2 cells without the pIgR expression did not allow transfer of IgM from the basolateral to the apical side of the Transwell cultures (data not shown). We further measured the transport capacity of HIV-1, primary isolates from clade B, and an HIV-1 Lai/IIIB laboratory strain^28^. All HIV-1 isolates penetrated the Caco-2-pIgR+ cell line and was transported to the basolateral side and HIV-1 p24 antigen became detectable within 1 hour post-challenge at the basolateral side of the Transwell chamber system (Fig. 2A). The transcytosed viral particles in the basolateral medium represented an infectious virus because it gave rise to infection when co-cultured either with Jurkat T cells or human activated PBMCs (Fig. 2B). Because seminal plasma and cervical secretion are known to contain both viral particles and infected cells^29–33^, we assessed the transcytosis efficacy when infected PBMCs were present at the apical side together with cell-free HIV-1. The addition of the HIV infected PBMCs enhanced the transcytosis for two of the HIV isolates namely Lai/IIIB and 6727^18^.

### Inhibition of transcytosis of infectious HIV-1 by human anti-HIV-1 env IgM monoclonal antibodies

**Figure 3:**
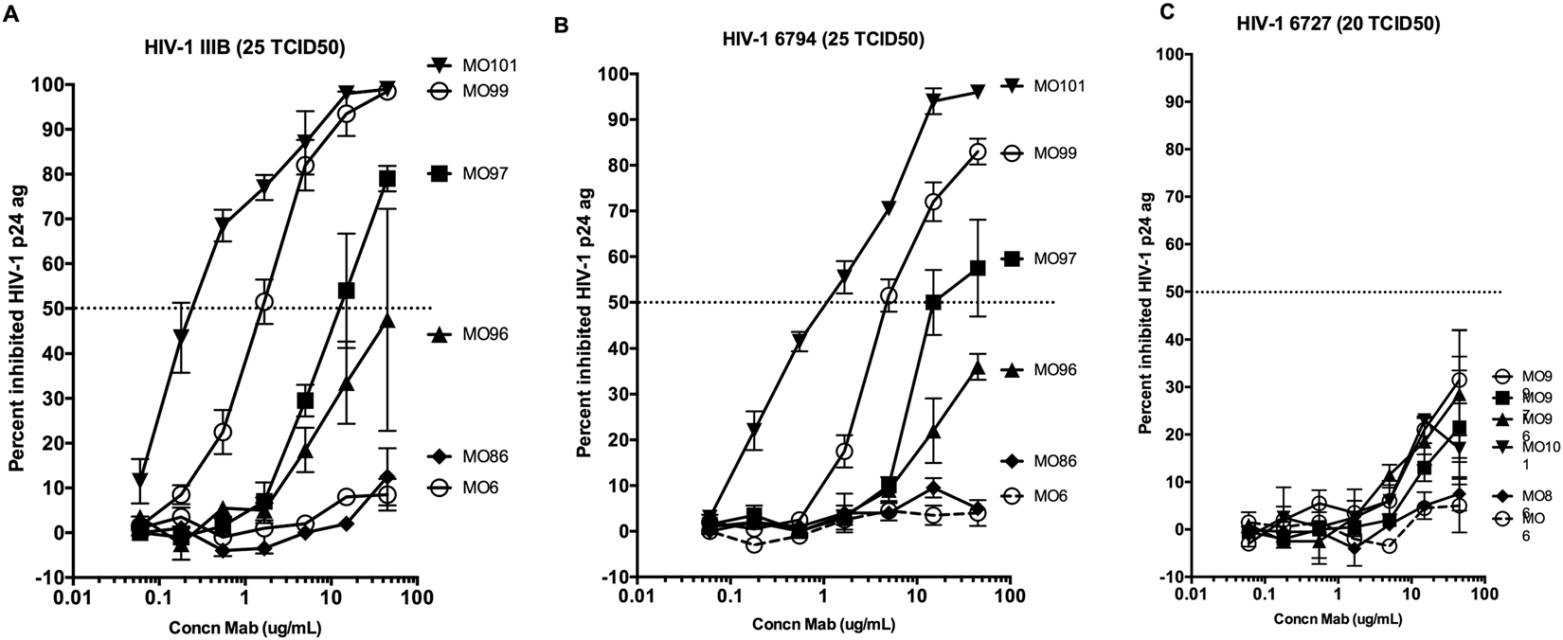
Neutralization of infectious cell-free HIV-1 with human IgM monoclonal antibodies and controls. The *in vitro* neutralizing Human anti-HIV-1 IgM MO101, MO97, and MO99, MO96 and the control non-neutralizing MO86 and MO6 with three HIV-1 isolates at equal virus concentrations (25 TCID59) (**A**) with HIV-1 IIIB in (**B**) with HIV-1 6794 isolate and in (**C**) with HIV-1 6727 isolate. The 50% inhibited HIV-1 p24 detection is indicated with the dotted line.

We assessed the ability of the human anti-HIV IgM MoAbs MO101, MO97, and MO99 to inhibit transcytosis of a syncytium inducing HIV-1 clade B primary isolate 6794 and HIV-1 Lai/IIIB. Supernatant from the basolateral sides of the Transwell chamber was collected 24 hours after exposure to virus and monoclonal antibodies. The epithelial cells in the Transwell chamber were washed, and the presence of infectious virus in the cells was determined using activated human PBMC as target cells. The MoAbs MO101 and MO99 significantly blocked the transcytosis at the basolateral side (91% and 89% p24 antigen reduction, respectively, p < 0.01) and within the cells (69% and 74% p24 antigen reduction, respectively, p < 0.01) as compared to HIV-1 with no antibody (Fig. 3A,B). The MoAb MO97 was less efficient in inhibiting HIV transcytosis (47% p24 antigen reduction, p = 0.15 n.s) compared to HIV-1 with no antibody present (Fig. 3B). Moreover, we observed no viral inhibition at any side of the polarized epithelium or in the cells when the MO101 was added in the presence of soluble rgp160, which neutralizes the MoAbs (Fig. 3A,B). The non-HIV neutralizing control MoAb MO6 did not significantly (range 16–25%) inhibit the transcytosis (Fig. 3A). A summary of the basolateral HIV-1-inhibiting properties of the HIV-1 envelope-specific MoAbs and negative controls are shown in Table 1. Inhibition of HIV-1 was not detected in our control experiments using human anti-Digoxin specific control MoAbs MO6 either alone or in combination with 10 µg of rgp160 **(**Table 1**)**.

**Table 1:** Kinetics of IgM Mab-binding to recombinant gp160 and inhibition of HIV-1 transcytosisacross Caco-2-IgR+ cells.

HIV-1 gp120 V3 specific	Time (h)	ELISA	Inhibition of HIV-1 transcytosis		
		Rgp160 binding (OD)*	HIV-1 IIIB (SI)**	HIV-1 6794 (SI)	HIV-1 6727 (NSI)
**monoclonal antibodies**					
MO96	0	0.010/0.008			
	14	0.140/0.111	87%/94%	90%/70%	25%/31%
	24	0.652/0.596	87%/94%/91%	90%/70%/76%	25%/31%/35%
MO97	0	0.009/0.010			
	14	0.151/0.148	23%/13%	38%/0%	1%/-5%
	24	0.497/0.483	23%/13%/26%	28%/10%/30%	1%/-5%/0%
MO99	0	0.011/0.012			
	14	0.201/0.209	83%/79%	68%/70%	41%/36%
	24	0.599/0.604	83%/79%/84%	88%/80%/91%	41%/36%/35%
MO101	0	0.014/0.011			
	14	0.166/0.144	88%/93%	91%/77%	5%/15%
	24	0.552/0.505	88%/93%/89%	91%/77%/90%	5%/15%/65
MO86	0	0.007/0.009			
	14	0.103/0.098	35%/36%	3%/4%	10%/13%
	24	0.393/0.402	35%/35%/36%	3%/4%/3%	11%/13%/9%
**Control IgM**					
**monoclonal antibody**					
MO6	0	0.016/0.009			
	14	0.020/0.011	44%/0%	11%/2%	18%/22%
	24	0.028/0.014	34%/0%/1%	11%/2%/0%	18%/22%/5%
Medium	0	0.014/0.010			
	14	0.020/0.012	0%/0%	1%/0%	0%/0%
	24	0.018/0.012	0%/0%/05	1%/0%/0%	0%/0%/0%

*Apical transwell culture medium collected and tested for anti-gp160 IgM binding activity at 0–24 hours by ELISA.

**Inhibition of HIV-1 p24 gag antigen in the basolateral transwell culture medium in the presence of anti-gp120 V3 Mabs compared with wells with control IgM or without antibodies.

### Inhibition of HIV-1 by human IgM monoclonal antibodies against HIV-1 envelope in human target cells ***in vitro***

**Figure 4:**
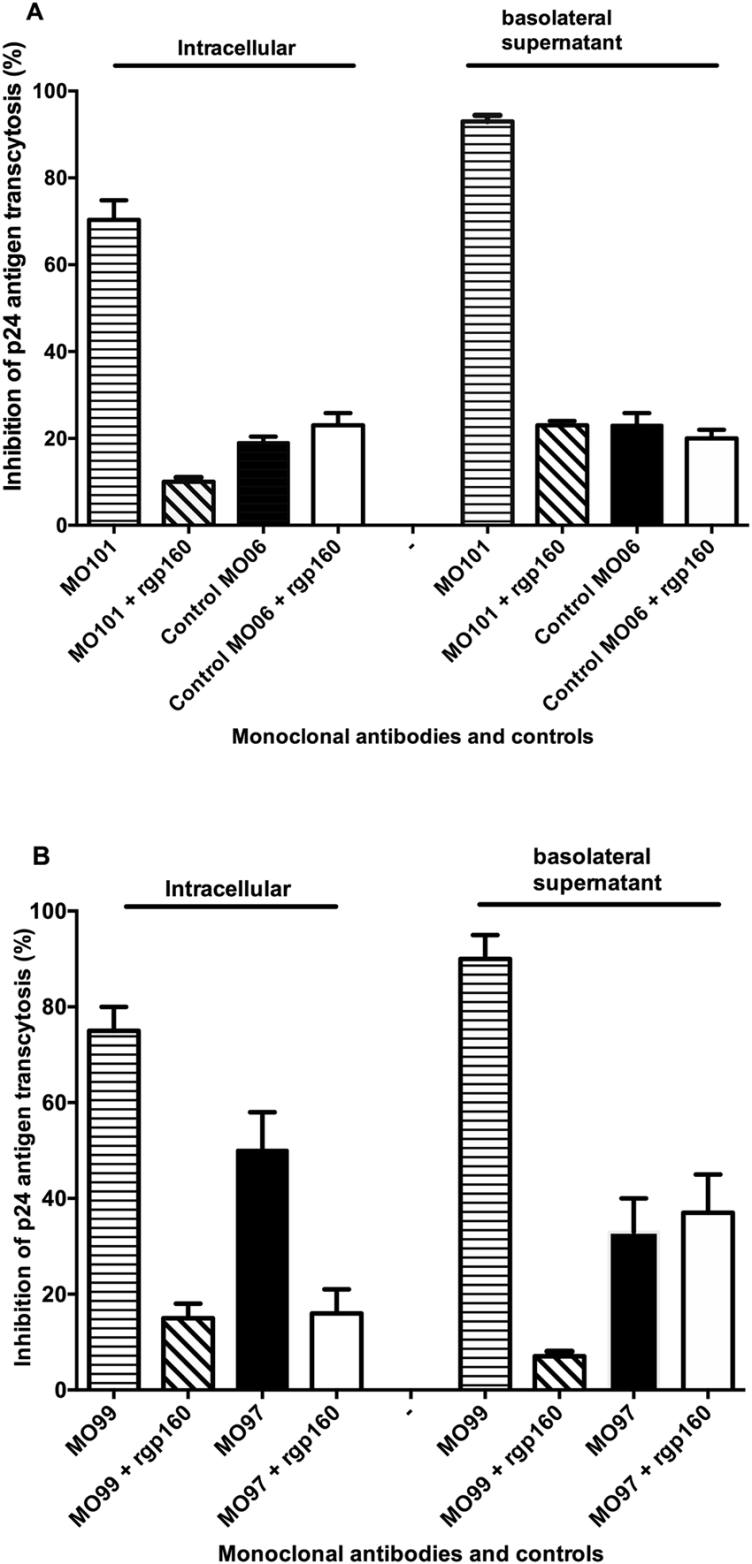
Inhibition of transcytosis of infectious HIV-1 by human anti-HIV-1 env IgM monoclonal antibodies. The *in vitro* neutralizing Human anti-HIV-1 IgM MO101, MO97, and MO99 and the control non-neutralizing MO6 with or without 5 µg/mL rgp160 were added together with HIV-1 SI 6794 infected PBMC during 24 h on the apical side to assess inhibition of HIV-1 transmission/transcytosis from the apical side to the basolateral side across the human epithelial cell line Caco-2-pIgR+. The inhibition of HIV transcytosis was assessed intracellularly in the cells and in the basolateral supernatant by p24 ELISA. (**A**) The *in vitro* neutralizing Human anti-HIV-1 IgM MO101 and the control non-neutralizing MO6 with or without rgp160 (p < 0.01), (**B**) the *in vitro* neutralizing anti-HIV-1 IgM MO99 (p < 0.01) and MO97 (p = 0.05) with or without rgp160. Mann Whitney U test was used to determine if the samples were significantly different than the negative control (**p < 0.01.and *p < 0.05). The bars represent the median percent and range inhibited HIV-1 p24 antigen *in vitro* from two to three repeated experiments. Comparisons of inhibited HIV-1 p24 were performed between control IgM (MO6) and the anti-HIV-1 neutralizing IgM MoAbs.

When the MoAbs were tested against free HIV-1 virus with human T cell line Jurkat T cells three MoAbs were shown capable of neutralizing two out of three HIV-1 isolates (Fig. 4A–C).

### Inhibition of HIV-1 by human IgM monoclonal antibodies against HIV-1 envelope in human cervical mucosa biopsies and transfer and infection to mucosal Dendritic cells

**Figure 5:**
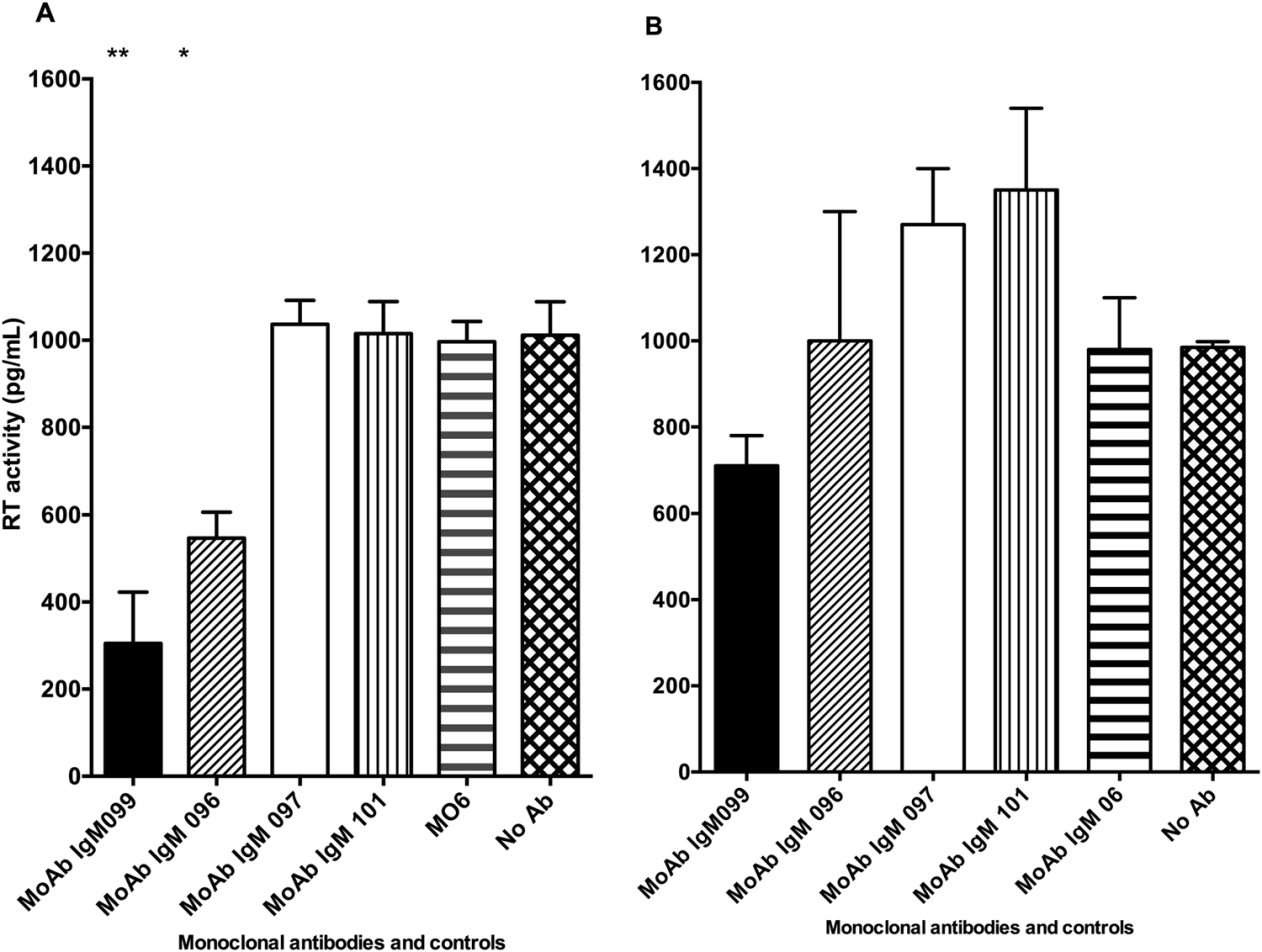
Inhibition of HIV-1 by human IgM monoclonal antibodies against HIV-1 envelope in human cervical mucosa biopsies and transfer and infection of dendritic cells. Inhibition of HIV-1 actively replicating (RT enzyme activity) from anti-HIV IgM-treated ectocervical biopsy tissue. (**A**) The quantitative RT activity (pg/ml) in the supernatant at the end of the study period. The bars illustrate the median and range from representative duplicate well inhibition assays. Mann Whitney U test was used to determine if the samples were significantly different than the negative control (**p < 0.01, *p < 0.05). (**B**). Figure 5B (**C**) The quantitative RT activity (pg/ml) in the supernatant at the end of the study period. The bars illustrate the median and range from representative duplicate well inhibition assays (**p < 0.01, *p < 0.05).

**Figure 6:**
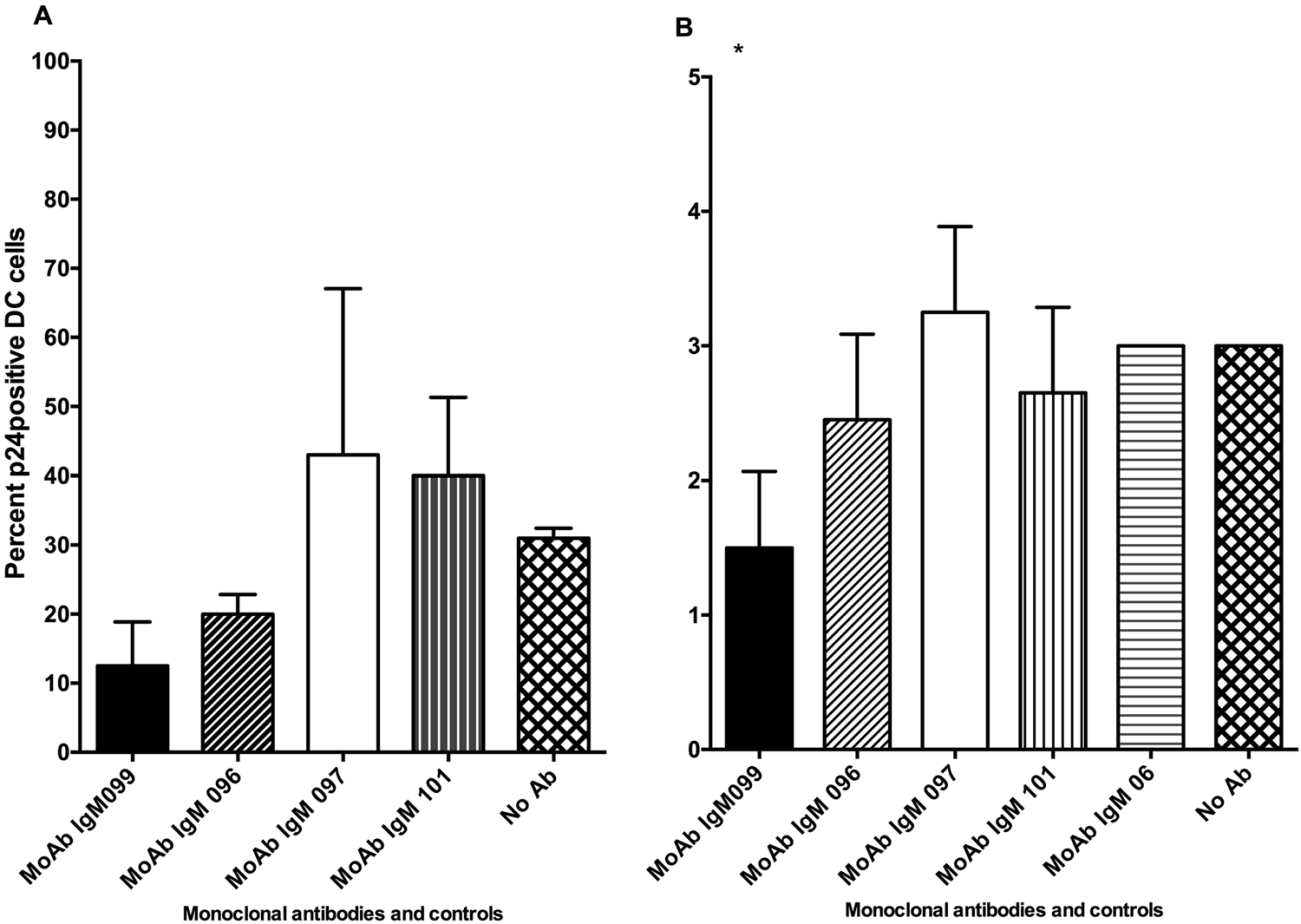
Effect of anti-HIV IgM antibody in cervix biopsies on the percentage of HIV-1 p24+ dendritic cells (DC). (**A**) The percentage of p24+ DCs in endocervical tissue and (**B**) the percentage of p24+ DCs in ectocervical tissue after 24 h of HIV-1 with and without monoclonal antibody exposure *in vitro*. The bars illustrate the range from duplicate inhibition assays. Mann Whitney U test was used to determine if the samples were significantly different than the negative control (*p =  <0.05).

To further investigate the HIV-1-inhibiting properties of the IgM MoAbs we tested their capacity to block HIV replication in primary mucosal cervical tissues. The two anti-HIV MoAbs MO99 and MO96 reduced the amount of RT-activity by 70% (range 62–89) respectively 46% (range 38–62%) in ectocervical tissue explants, whereas no inhibition was observed with the MoAbs MO97 and MO101 or with control antibodies (Fig. 5A). In endocervical tissue explants, only MoAb MO99 succeeded in reducing the RT-activity, (28%, range 26–34%) (Fig. 5B), compared to tissues without antibody. To confirm the reduced HIV-1 RT in the supernatant from the cervical tissue explants exposed to MoAbs MO99, MO96, MO97, and MO101 we assessed the HIV-1 infection of emigrating cervical DCs by staining for HIV-1 p24. The MoAb MO99 reduced the percentage of HIV infected emigrating DCs from both endo and ectocervix, with significant reduction for the endocervical DCs (Fig. 6A,B).

## Discussion

It is well known that the presence of early neutralizing antibodies during an infection can protect against lethal disease^34–36^. IgM is the first immunoglobulin produced during a primary infection and can be transported through polarized mucosal tissues, thus providing a first layer of protection against invading pathogens^37–41^. In some instances, it has been shown that IgM to have low affinity but high avidity with a broad antigen recognition capacity due to its multiple epitope-binding properties^6^. Furthermore, IgM can interfere or inhibit bacterial and viral attachment and also viral infection of cells and tissues^37–43^. Interestingly, even non-neutralizing IgM and IgG show viral infection-reducing properties and this has been reported for Marburg and Ebolavirus^43^. For this reason, we evaluated whether anti-HIV IgM monoclonal antibodies were capable of inhibiting HIV-1 transcytosis through a polarized epithelial cell barrier *in vitro*. We demonstrated, in an intestinal epithelial cell line expressing pIgR, that the antibodies were transported from the basolateral side to the apical surface. The pIgR expressing epithelial cells also allowed the transport of infectious HIV-1 from the apical to the basolateral side of the polarized epithelium. We also showed that two human IgM monoclonal antibodies, MO96 and MO101, directed against the V3 loop region of HIV-1 gp120 inhibited HIV-1 transcytosis across the epithelial cell layer. This inhibition was observed when using an HIV-1 primary isolate of the SI phenotype and with a laboratory-adapted HIV-1 isolate. However, when the epithelial cells were challenged with the HIV-1 primary isolate of the NSI phenotype, no inhibition of the transcytosis was observed. This indicates that IgM epitope recognition of the transcytosed HIV-isolates is important and that the V3 region may be better exposed on the SI strains than on the NSI isolates. Primary isolates may have a more closed exposure of the outer envelope epitopes than T-cell line adapted HIV isolates^44^. It was also possible to reduce the antiviral activity of MoAbs transcytosis by blocking with V3 peptides or rgp160 protein representing SI isolates, thus indicating the need for epitope specificity of the antibodies.

Interestingly, the monoclonal IgM antibodies with HIV-1 envelope specificity had some *ex vivo* efficacy in reducing HIV-1 replication and intracellular entry into antigen-presenting cells, i.e. dendritic cells (DCs), in primary human cervical tissue samples. There was some degree of variability between the IgM MoAbs in their capacity to inhibit HIV-1 replication in ectocervical and endocervical tissues. The differences in their efficacy are not yet clear and it is important to note that the IgM concentrations used were not very high and spanned from 5 to 9 µg/mL. Another factor that may play a significant role is the difference in the IgM avidity against the HIV-1 envelope epitopes, as well as the gp120-V3 epitope specificities between the MoAbs previously described^20^. Furthermore, a variation in the numbers and activation stages of the HIV-1-susceptible cells in the cervical issue biopsies is also to consider. However, even with these assay limitations, an interesting result concerning the numbers of HIV-1 p24-positive DCs detected in the endo- and ecto-cervical biopsy tissues was observed in the presence of some of the IgM MoAbs. Depending on whether the tissue was collected from the endo- or ectocervix, one or two of the IgM antibodies showed a reducing effect on the number of p24-positive DCs. This promising data obtained from the primary cervical tissues further indicate IgM anti-HIV-inhibiting properties.

Secretory IgA and IgM antibodies can be efficiently transcytosed and may function as the first specific barrier against infection^19,27,44^. However, polymeric IgA is more efficiently transcytosed than polymeric IgM^44,45^. Virus-neutralizing antibodies in mucosal surfaces, such as interactions between IgA and influenza or Sendai viral envelope proteins and intracellular neutralization of influenza virus have been previously reported^15,44^. In this study, we investigated whether the neutralization of HIV occurred at the intracellular or at the apical surface of the cell line. We could see the intracellular reduction of the p24 content with the addition of HIV-1-neutralizing IgM MoAbs, which is accordance with Bomsel *et al*.^16^. At best, approximately 6 × 10^11^ IgM molecules in a 24 hour period could be transferred to the apical side of the cell line (data not shown). In the pIgR-transfected cells, these values were 2.5 × 10^11^ monoclonal IgM molecules per 20 hours. If each Ig-molecule has the capacity to neutralize one free HIV particle and if this epitope is available at the HIV-exposed mucosal surface, a high amount of neutralizing HIV-1-specific antibodies could be transported. Thus, as long as the antibodies can efficiently recognize the invading virus, this should be an opportunity for the immune defence to deliver a specific, sterilizing immunity. The effect of HIV-neutralizing antibodies on invading HIV-1-infected cells is more difficult to predict. Notably, both antibodies MO96 and MO101 were raised from B-lymphocytes obtained from a healthy, uninfected blood donor, clearly demonstrating that the normal human B-lymphocyte repertoire produce antibodies that are capable of interfering with HIV infection.

Several studies have described the importance of cell-mediated immunity as a primary response to HIV-1 infection^46,47^. The results of our study suggest a potential important role for nIgM immune mechanisms in the mucosal compartment. It is possible that an epithelium that is sufficiently efficient to allow the transcytosis across epithelial cells of IgM with high to moderate avidity to the HIV-1 envelope and that the pIgR may protect against infection. Several non-specific defence mechanisms such as released lysozomes and antimicrobial peptides such as defensins and innate nitric oxide are present in epithelial cells and at mucosal surfaces^48–51^. Normally, they protect us from many infections, but when the primary defence barrier fails or a microbiological breakthrough occurs, specific immunity, such as immune memory B-cells secreting sIgA or sIgM are invaluable to the host.

Additionally, natural IgM antibodies may also be present in early immune responses as a first line of defence against bacteria and viruses^52–56^. These antibodies promote the formation of the immune complexes that are required to generate a germination centre in which conventional B-cells can mature and produce high affinity antibodies. It has been postulated that the binding of virus particles with sIgM may reduce the viral infectivity intracellularly^16,44^ and may lead to complement activation to facilitate viral clearance by phagocytic cells^57,58^. It has been shown that sIgM can confer protection by promoting an efficient antiviral IgG response both natural and specific IgM are required for the induction of IgG responses^54,56,58^.

The transmission of HIV-1 can occur via exposure of the genital, rectal or mucosa to infected seminal fluids^55,59^. Some studies have reported that epithelial cells are not capable of being infected, thus allowing the active transport of viruses and immunoglobulins to the submucosa layer, other studies have shown that human colonic epithelial and vaginal epithelial cells can be infected with HIV-1^56,59^. It has been shown that HIV-1 infection of epithelial cells was neutralized by antisera against three conserved regions and the V3 loop located on the gp120^18,44,56^.

We have previously shown that sIgA specific for binding HIV-1 envelope proteins from highly exposed seronegative individuals (HESN) can block HIV-1 transcytosis across the mucosal epithelium^18^. In this study, we have demonstrated that IgM produced from a normal blood donor could be transported across these epithelial cells in concentrations sufficient to prevent the transmission of HIV particles and viral dissemination. We could contemplate the possibility that immediately after HIV entry interactions between nIgM and HIV occur that shape the subsequent immune responses and protect HEPS individuals from infection. Thus, the presence of natural IgM maintains mucosal equilibrium by clearance of apoptotic cells, altered cells by macrophages, dendritic and B-cells and the role in protection against infection was seen in the neutralization, opsonization and recognition of HIV, B-cell isotype class-switching and induction of memory B-cells. However, even though B-1 cells have a special property for self-renewal and there is a continuous IgM production during life, some patients developed a selective loss of natural IgM that leaves them vulnerable to infections. Investigations circumventing the loss of local immunoglobulin immunity have focussed on restoring IgM production via the administration of IL-18^58^, and the use of monoclonal IgM in experimental autoimmune disease^60^, but to date these issues have been evaluated only in a few disorders^58^.

To summarize, our data clearly showed that the specific HIV-1 blocking and neutralizing *in vitro* properties and these data, together with previous studies^16,19,27^, support the hypothesis that natural IgM antibodies may be one of several contributing factors in limiting the initial susceptibility to HIV-1 infection via mucosal exposure. If the exposure is at a low infectious dose, even these low concentrations of natural IgM or IgA antibodies may have some preventive effects. Together with other innate factors, such as RANTES, MIP-1/-2 and anti-microbial peptides, they may act as additives or even as synergistic anti-HIV-1 molecules *in vivo*. We suggest that natural IgM in mucosal epithelial tissues may participate as a potential contributor in the prevention against infection, such as HIV-1. However, the extent of its contribution still need to be better understood and further investigated.

## Material and Methods

### Human monoclonal antibodies

Human monoclonal antibodies of the IgM isotype specific for the third variable (V3) loop region of the envelope of the HIV-1/LaiIIIB were developed and produced as previously described^20,61^. Briefly, B-lymphocytes from HIV-1 negative donors were immunized *in vitro* with recombinant protein pB1, covering amino acid residues 286–467 of the HIV-1/Lai Gp120 (kindly provided by Repligen Corp, Cambridge, MA, USA). After six days of immunization with 25–250 ng pB1/mL, the lymphocytes were immortalized by Epstein-Barr virus infection. Cell lines secreting antigen-specific human IgM MoAbs were detected by an antigen-specific ELISA as described previously^20,61^. In the transcytosis assay, 10-fold concentrated hybridoma supernatants of clones MO96, MO97, MO99 and MO101 specific for HIV-1 V3 and non-HIV binding control IgM clones MO86 and MO6 were used. The Mo86 IgM clone recognizes an epitope outside of the V3 loop and it has been shown not to neutralize infection with HIV-1 and it does not bind HIV-1-infected cells^20^ and the MO6 IgM clone is directed against digoxin^62^. All antibodies were developed from blood from HIV-1 seronegative individuals obtained from the University hospital blood bank (Lund, Sweden). Studies were performed with the approval of the ethical review board at The Lund University hospital. All blood was obtained with informed consent according with the Declaration of Helsinki.

### Immunoassay (ELISA)

Ninety-six well plates (NUNC, Aahus, Denmark) were coated with 100 ng/well of pB1 (Repligen, *Cambridge, MA*) or baculovirus recombinant gp160 (Protein Sciences, Meriden, CT, USA) in 100 μl NaCO_3_ buffer (pH 9.6). Samples containing MoAbs were diluted in 10 mM sodium phosphate buffer (pH 8.0, 0.15 M NaCl, 0.5% BSA and 0.1% Tween 20). The hybridoma supernatant culture medium was diluted and added at 100 µl/well into antigen-coated plates that had previously been washed with NaCl/Tween 20. These plates were incubated at 37 °C for 60 min and horse-radish peroxidase (HRP)-labelled goat anti-human IgM (Zymed Labs, San Francisco, CA, USA) was added as a conjugate at a 1:4000 dilution. A one-hour incubation was repeated, plates were washed and TMB-substrate (Sigma, St. Louis, MO, USA) was added at 100 µl/well. The substrate reaction was stopped by the addition of 100 µl of 2 M H_2_SO_4_ and the absorbance was measured at 450 nm.

### Polarized epithelium cell culture

To measure the transport of the IgM monoclonal antibodies from the basolateral to the apical side, a polarized epithelial cell line, the human intestinal epithelial cell line Caco-2, transfected with pIgR cDNA, was used. Comparison was performed with non-transfected Caco-2 cells, where no IgM transcytosis was seen (data not shown). Analysis of HIV-1 transcytosis from the apical to the basolateral side was analysed in the human Caco-2-pIgR+ cell line. The cell culture was performed on Transwell nitrocellulose filters (3-µm pore size filters, Costar, Cambridge, MA, USA) in MEM containing 10% inactivated foetal calf serum (Gibco, Life Technologies, Scotland) for 8–10 days, until the cell lines formed tight monolayer cultures. One millilitre of medium/well exchange at the basolateral side and 0.5 mL/well at the apical side was collected and exchanged every 72 hours. The tightness of the epithelial cell was measured as electrical resistance of >400 Ohms/cm^2^ for the Caco-2 cells (range 420–630 Ohm). Transepithelial electrical resistance was measured using a Millicell RS resistance apparatus (Millipore, Bedford, MA, USA) as described elsewhere^18,63^.

### Transcytosis of human IgM antibodies

Medium containing human IgM MoAbs (5–9 µg/ml) was added to the basolateral side of the Transwell chamber. Fifty microlitre of medium/chamber side was collected from the apical side after 0, 1, 2, 4, 6, 12, 48, and 72 hours and analysed by ELISA for the content of IgM. The amounts of IgM were analysed by capture-ELISA using goat-anti human IgM-specific reagents (Vector, Burlingame, CA, USA). A standard of purified human IgM was used in the ELISA (Sigma-Aldrich, St. Louis, MO, USA) as a positive control. Statistical analyses were performed with non-parametric Mann-Whitney U tests and student’s t-tests with the Prism 6 for Mac OS X (GraphPad Software MacKiev, CA, USA).

### HIV-1 neutralization assay

HIV-1 neutralization *in vitro* was performed as previously described^20^. In brief, HIV-1 isolates at 25 TCID50/mL concentration was added to serially diluted IgM MoAb concentrations (45-0, 1 ug/mL) and control samples in triplicate wells diluted in RPMI 1640 medium with 10% FCS, supplemented with 1% penicillin-streptomycin (GIBCO Life Sciences, Paisley, Scotland), in 96 well tissue culture plates (200 µL/well) and incubated for 1 h at 37 °C. Cells were added, 100 000 cells/well in 50 µl/well and incubated 1 h. Cells were washed twice with 200 uL RPMI 1640 medium/well by centrifugation and cells were then cultured for 6 days in complete RPMI 1640-10% FCS medium before HIV-1 p24 quantitative ELISA was performed as described below.

### Transcytosis of HIV-1 isolates, HIV-1 isolation and HIV-1 blocking

Three different HIV-1 isolates were used to measure HIV-1 transcytosis, one T-cell line adapted strain Lai/IIIB^28^, and two primary HIV-1 isolates. The two primary HIV-1 clade B isolates represented different phenotypes, the syncytium-inducing (SI) isolate 6794 and the non-syncytium inducing (NSI) isolate 6727 (Swedish Institute for Infectious Disease Control, Stockholm, Sweden). Six hours after the basolateral addition of IgM, 1.5 mL of the isolates 6727 or 6794 as well as the HIV-1 Lai/IIIB isolate was mixed with 100.000 infected PBMCs and added to the apical side of the chamber to analyse the virus transcytosis and basolateral inhibitory capacity of the human IgM monoclonal antibodies. Each HIV-1 isolate infected PBMC with virus mixture was tested in separate wells, by incubating HIV-containing supernatant mixed with target cells for 24 h at +37 °C and thereafter washed with RPMI 1640 medium for continued virus cell culture. Supernatant (100 µl) from the basolateral side was collected after 0, 1, 2, 4, 6, 12, 24, 48 and 72 h, and their p24 antigen content was measured in a p24 capture ELISA^64–66^. Furthermore, 100 µl of medium collected from the basolateral side was collected after 0, 1, 2, 4, 6, 12, 24, 48 and 72 h and used for HIV-1 isolation analyses to investigate if the transcytosed viruses were infectious. The collected basolateral medium was added to one million PHA/IL-2 activated human peripheral blood lymphocytes in 1 ml of cell culture volume *in vitro*. Collected basolateral virus-containing medium and cells were cultured for 48 h at 37 °C in 5% CO_2_/air and thereafter washed with RPMI 1640 supplemented with 1% penicillin-streptomycin (GIBCO Life Sciences, Paisley, Scotland), 10% inactivated foetal calf serum, and 200 IU/ml recombinant interleukin-2 (Amersham, Uppsala, Sweden). The presence of intracellular infectious virus and the inhibitory capacity of the monoclonal antibodies was measured by using the following procedure: wells containing the Caco-2-pIgR+ cells were washed on both sides three times for 5 min with serum-free medium (37 °C). Phosphate-buffered saline (500 µl) with 1% trypsin was added to each well, and trypsinization was carried out in Eagle’s minimal medium supplemented with 5% FCS. Cells were spun down and used for HIV-1 virus isolation. HIV cell culture was performed for 2 weeks with 10^6^ PHA-IL2-activated PBMCs in a mixed lymphocyte culture to measure the presence of viable HIV. The reduction of >67% of the HIV-1 p24 antigen content in the culture medium compared with the control samples without antibodies was considered a positive blocking effect.

To control the tightness of the epithelial cell barrier, 1.5 mL of recombinant p24 antigen (vector) or gp160 antigen (1 µl/mL) mixed with medium was added to the apical side and the presence of the recombinant antigens on the basolateral side was analysed after 1–48 hours (data not shown). This integrity and tightness of the polarized epithelial cells was performed using the Millicell RS resistance apparatus (Millipore, Bedford, MA) and shown to be 400 (range 380–444) mOhm. Statistical analyses were performed with non-parametric Mann-Whitney U tests and Student’s t-tests with the Prism GraphPad Software, CA. Human peripheral blood mononuclear cells were obtained from the Karolinska University hospital blood bank (Solna, Sweden). Approval was given by the Karolinska Institute institutional review board for these studies. Informed consent was obtained in accordance with the Declaration of Helsinki.

### Cervical tissue sampling, monoclonal antibody treatment and virus inoculations

Cervical tissue was obtained from healthy women undergoing prolapse surgery at the Linköping University Hospital (Ethical permit EPN M206-06)^67^. This collection and study of biopsies was approved by Linköping Ethical Review board and informed consent from all participants was obtained. All methods were carried out with relevant guidelines and regulations at the Linköping University hospital. We confirm that all experimental protocols were approved by the ethical review board of Linköping University hospital.

Cervical samples containing both endo- and/or ectocervical tissues were kept on ice and processed within 30 min after removal. The epithelial layer and lamina propria were separated from the underlying stroma, and explants of approximately 6 mm^2^ were placed in culture plates containing RPMI 1640 containing 20 µg/mL Gentamicin, 10 mM HEPES (Fischer Scientific, Gothenburg, Sweden) and 5% pooled HS (Fisher Scientific). Cervical explants were preincubated for 30 min at 37 °C for treatment with the different monoclonal antibodies (5 µg/ml) as described for other inhibitors^66,67^. The tissue explant cultures were infected with HIV-1 primary isolates (0.5 µg p24/ml) and incubated for 2 hours at 37 °C and washed four times and transferred to six-well tissue culture plates. After four days, cells were collected, stained for CD3, CD4, CD1a, and p24 and subjected to fluorescence-activated cell sorting (FACS) analysis. To compare results obtained from separate experiments, using cervical explants derived from tissue donors, the HIV infection of emigrating DCs and CD4+ T cells in the absence of monoclonal antibodies was normalized to 100% for each experiment. Infected cell explants were fixed in 4% paraformaldehyde (PFA) and stored for immunohistochemistry and /or immunofluorescence staining. The presence of active HIV-1 virus was analysed with the HIV-1 reverse transcriptase activity ELISA according to the manufacturer’s instructions (Caviditech, Uppsala, Sweden).

### Statistical analyses

Statistical comparisons were performed using the Graphpad Prism 6, Graphpad InStat (version 6.1a 2015 Software Inc. San Diego, CA, USA). Non-parametrical comparisons between negative control IgM MoAbs and each of the HIV-1 specific IgM specific MoAbs were performed with the Mann Whitney U test. A p-value of <0.05% was considered significant, and ns indicates non- significant.

### Data Availability

The datasets generated during and/or analyzed during the current study are available from the corresponding author on reasonable request.
